# Dr. Elizabeth Blackwell (1821 - 1910): Opening Doors to Women in Medicine

**DOI:** 10.7759/cureus.71899

**Published:** 2024-10-20

**Authors:** Sandhya Nallamotu, Arundhati Vankayalapati, Sreenija Paruchuri

**Affiliations:** 1 General Medicine, Kasturba Medical College, Manipal, Manipal, IND; 2 General Medicine, Methodist Richardson Medical Center, Richardson, Texas, USA; 3 Orthopaedic Surgery, Nizam's Institute of Medical Sciences, Hyderabad, IND; 4 Orthopaedic Surgery, Kamineni Institute of Medical Sciences, Narketpally, IND

**Keywords:** female physician, gender equality, general practice, historical vignette, medical education, obstetrics and gynecology, preventive & social medicine, uk, usa, women's rights

## Abstract

Dr. Elizabeth Blackwell spearheaded the role of women in the medical field as physicians. Most women of the time took up nursing or practiced medicine without formal educational qualifications. However, she challenged the gender roles within the medical field to show the world that women can excel in the study of medicine, graduate with a medical degree, and contribute as physicians in the community on par with their male counterparts. When garnering equal respect in the field despite holding a valid medical license and degree proved difficult, she opened the doors for other women pursuing medical education by founding medical colleges for women. During her lifetime, she was a global physician. Since she graduated from medical school, women have gradually found equal footing with men in the medical physician community. The impact women have on the medical field is undeniable; not only are they nurses and paramedical professionals, but women make up some of the top physicians and surgeons around the world today. This biographic account of the life and career of Dr. Elizabeth Blackwell aims to honor her memory in the advancement of gender equality within the medical field for women.

## Introduction and background

Dr. Elizabeth Blackwell established equality of gender within the medical field. Throughout her life, she fought for women's rights, advocated for the poor, and established the path for women to become Doctors of Medicine globally [1]. She was the first woman to be accepted into Medical College, and graduate to receive a Doctor of Medicine (MD) in the United States of America [2]. With the combined efforts of her sister Dr. Emily Blackwell and a colleague Dr. Marie Zakrzewska, she started the Women’s Medical College of the New York Infirmary. It is the first four-year medical college in the US, and today continues to run under Weill Cornell Medicine as the New York Presbyterian Lower Manhattan Hospital [3-5]. During the American Civil War, she played a vital role in setting up a training association to help prepare nurses for the war efforts [3]. In the United Kingdom, with a strong reference from her friend Ms. Florence Nightingale, she became the first woman to be registered with the British Medical Registrar [6]. She also helped found the National Health Society and open the first medical college for women in London - the London School of Medicine for Women [3]. Dr. Elizabeth Blackwell opened the doors of medical science to women across the USA and Europe.

## Review

Early Life

Born on February 3, 1821 in Bristol, England, Ms. Elizabeth Blackwell was the third of Samuel Blackwell and Hannah Lane's nine children. Her father was a sugar refiner and a Quaker, passionate about social reform, including anti-slavery activism, thereby instilling a strong sense of justice and education in his children. The Blackwell family migrated to the United States in 1832, settling first in Cincinnati, Ohio, where Samuel sought better economic opportunities. Unfortunately, in 1838, he died, leaving the family struggling financially during a broader national economic crisis [1,7].

Ms. Elizabeth Blackwell and her mother, alongside her older sisters, worked as teachers to support the family, emphasizing the educational values instilled in them from an early age. Despite their financial challenges, the Blackwell family remained committed to education, and Ms. Elizabeth Blackwell was encouraged to pursue her interests, particularly in subjects deemed suitable for women of her time. This environment of intellectual curiosity and advocacy for women’s rights would later shape her determination to enter the medical field [8-10]. 

Discovery of medicine as a vocation

While teaching, Ms. Elizabeth Blackwell was greatly influenced by her friend Mary Donaldson. Ms. Donaldson, who was dying of cancer, expressed great distress over the lack of female physicians to treat her. Ms. Elizabeth Blackwell recounts in her memoir, *Pioneer Work in Opening the Medical Profession to Women*, how Ms. Donaldson asked her, “You are fond of study, have health and leisure; why not study medicine? If I could have been treated by a lady doctor, my worst sufferings would have been spared me” [11]. These poignant words prompted Ms. Elizabeth Blackwell to consider a medical career, believing that women could offer care that was more empathetic and relevant to other women’s unique medical needs [12]. Furthermore, after learning of the disadvantages and trials of marriage according to the societal norms of the time, Ms. Elizabeth Blackwell was keen on the life of a career woman to escape marital binds [11].

Initially, Ms. Elizabeth Blackwell faced skepticism from friends and family regarding her ambitions. Nonetheless, her desire to pursue medicine solidified into a moral imperative, united with her belief in women's right to equal opportunities in education. Determined to overcome societal barriers, Ms. Elizabeth Blackwell spent a year teaching music to save money for medical school, all the while studying medical textbooks in her spare time to prepare for her future [8,11].

Rejection and acceptance into medical school

In 1846, Ms. Elizabeth Blackwell applied to multiple medical schools in New England but was met exclusively with rejection. These experiences fueled her resolve to become a physician, leading her to correspond with notable physicians to seek their support for her goals. One of these doctors, Dr. Joseph Warrington in Philadelphia, although skeptical, encouraged her to pursue her dreams and offered to help by providing access to his medical library and lectures. He wrote to her stating, “If the project be of divine origin and appointment, will sooner or later be accomplished.” She would even be allowed to attend house calls and see patients alongside Dr. Warrington. When it was time for her to apply to medical colleges, Dr. Warrington, thoroughly impressed by Ms. Elizabeth Blackwell’s earnest efforts and interest in the profession, offered to write her a letter of recommendation to attend medical school [9,13,14].

After a year of persistent efforts and several more applications, Ms. Elizabeth Blackwell applied to Geneva Medical College in New York with an outstanding letter of recommendation from Dr. Warrington. Dean, Dr. Christopher Lee was shocked to see the first female applicant to his medical school. Following popular sentiment, he was not in favor of Ms. Elizabeth Blackwell, a young woman, studying Medicine. However, wary of offending Dr. Warrington, a highly respected physician of the time, Dr. Lee took a circumspect path to her application’s rejection. Instead of simply rejecting Elizabeth's application, he and his faculty decided to allow their medical students to vote on whether to accept her application, with the assumed confidence that the all-male student body would surely vote her out. Her application would only be accepted if all 150 male students voted to accept her application. Much to the astonishment of Dr. Lee and college faculty members every student voted in favor of her acceptance, having believed that the proposition was merely a practical joke. Unsurprisingly, her application was accepted under unusual circumstances.

Mass ridicule of her bold application led to its unassumed acceptance, nevertheless making her the first woman to be admitted to a medical college in the United States, a decision that would set a precedent for women's education in medicine [12]. Elizabeth held her admission letter to medical school to be one of her most precious possessions in life.

Her letter of acceptance to Geneva University read as follows: 

"Geneva: October 20, 1847.

To Elizabeth Blackwell, Philadelphia.

I am instructed by the faculty of the medical department of Geneva University to acknowledge receipt of yours of 3rd inst. A quorum of the faculty assembled last evening for the first time during the session, and it was thought important to submit your proposal to the class (of students), who have had a meeting this day, and acted entirely on their own behalf, without any interference on the part of the faculty. I send you the result of their deliberations, and need only add that there are no fears but that you can, by judicious management, not only 'disarm criticism,' but elevate yourself without detracting in the least from the dignity of the profession.

Wishing you success in your undertaking, which some may deem bold in the present state of society, I subscribe myself,

Yours respectfully,

CHARLES A. LEE, Dean of the Faculty.

At a meeting of the entire medical class of Geneva Medical College, held this day, October 20, 1847, the following resolutions were unanimously adopted:-

1. Resolved - That one of the radical principles of a Republican Government is the universal education of both sexes; that to every branch of scientific education the door should be open equally to all; that the application of Elizabeth Blackwell to become a member of our class meets our entire approbation; and in extending our unanimous invitation we pledge ourselves that no conduct of ours shall cause her to regret her attendance at this institution." [3]

Medical school

Ms. Elizabeth Blackwell arrived at Geneva Medical College in November 1847. Her first day was marked by apprehension, as her acceptance created a stir among the male students and faculty who had never before experienced a female classmate. Although she faced immediate challenges, such as exclusion from dissections and condescension from faculty and students alike, she remained steadfast in her commitment to her studies. [2, 14].

Despite such hurdles, Elizabeth excelled academically. Her dedication and intelligence gradually earned her respect among her peers. Committed to medicine, she followed a methodical and unfaultable approach to overcoming gender biases in its study. ​On January 29, 1849, Elizabeth Blackwell graduated at the top of her class, finally achieving her long-held dream of becoming a physician.​ Today, Geneva Medical College is renamed Hobart and William Smith Colleges. 

Her brother Henry Browne Blackwell recounted her graduation day in a letter: “He (the Dean of Geneva Medical College, Charles A. Lee) had pronounced her the leader of her class; stated that she had passed through a thorough course in every department, slighting none . . . and by her ladylike and dignified deportment had proved that the strongest intellect and nerve and the most untiring perseverance were compatible with the softest attribute of feminine delicacy and grace." This momentous occasion was celebrated widely, drawing public attention and marking a significant milestone toward gender equality in medicine [2].

Postgraduation

After graduation, she was not permitted positions for clinical training at any US hospitals. With the hope of gaining her clinical training at the well-established medical institutes and hospitals of Europe, she traveled to Paris. Much to her dismay, she was again barred from attending lectures in the European institutes on the basis of her sex. Only male members were admitted. A teaching hospital for midwifery training, La Maternité, was the only opportunity for clinical training availed to her. She was treated no differently from uneducated French village girls training in midwifery and found that her MD was of little value. Undeterred, she notes having learned a great deal about women’s and children’s health through the clinical art and perils of midwifery, making the most of the opportunity afforded to her at La Maternité. While treating an infant with ophthalmia neonatorum, the most common cause of childhood blindness at the time, the contaminated washings accidentally splashed into her eye. Despite the best medical care, she tragically lost all sight in her left eye. Dr. Elizabeth Blackwell was forced to give up all hopes of practicing surgery due to her severely compromised vision.

An avant-garde woman of science, Dr. Elizabeth Blackwell was cognizant of the limitations of allopathic medical practice. In pursuit of healing her vision, she explored and self-tested the abilities of alternative medical therapy such as mesmerism, homeopathy, and hydropathy. She found her analysis of these practices to be lacking in the ability to provide adequate relief from disease, eventually choosing to adhere to medical practice within the scope of her medical degree.

Later, she continued her studies at St. Bartholomew’s Hospital in London where she was greeted kindly by all the faculty except, in a twist of fate, the Professor of Midwifery. He openly voiced his disapproval of her plight to pursue medical practice as a woman. Paying no heed to his negative input, Dr. Elizabeth Blackwell continued her studies with a focused dedication toward her goal of practicing medicine to prove that women were capable of excellence within the field of medicine, at an equal footing to men. While in Europe, Dr. Elizabeth Blackwell met Ms. Florence Nightingale. Both women had similar backgrounds and many common interests, becoming quick friends. After a few months of studying in London, Dr. Elizabeth Blackwell returned to the USA [6,15].

Early career

In 1851 Dr. Elizabeth Blackwell moved back to New York City, ready to open her medical practice. Few patients sought her care and her employment was denied at established medical practices. The societal bias and antagonistic beliefs against a female physician were far from broken by her mere acquisition of an MD. Sexist views in society at the time were pervasive in the context of medicine. With a resolute spirit, Dr. Elizabeth Blackwell identified that poor women and children of New York City (NYC) were neglected adequate access to healthcare. To address this problem, two days a week, she opened a small clinic to provide care to the underserved communities of NYC, free of charge. Always punctual and steadfast in her medical duties, she was a pillar of support to the poor. She was accustomed to managing difficult circumstances in life and the lack of financial gains did little to deter her spirits. A great part of her work at the clinic centered around promoting hygiene and preventive medicine [16]. Today preventive medical practices are notably highlighted in preventive medicine and primary care.

Soon Dr. Elizabeth Blackwell's younger sister, Emily Blackwell, followed in her steps to receive her medical degree from Case Western Reserve University in 1854, becoming the third woman in the USA to earn a medical degree. Together, Dr. Elizabeth and Dr. Emily Blackwell, and another female colleague, Dr. Marie Zakrzewska started the New York Infirmary for Indigent Women and Children on May 12, 1857 [3, 6]. In 1862, *The New York Times* commented on the Infirmary explaining to its readers that "The objects of the Institution are to secure to poor women the medical advice of competent physicians of their own sex; to train nurses for the sick, and to assist well-educated women, who are studying medicine, in acquiring a practical knowledge of their profession." The Infirmary was staffed and run entirely by women. Financial subscriptions and donations supported the Infirmary. This was the first institution of its kind in the United States and abroad [4]. They launched a “sanitary visitor” program, a door-to-door service by medical professionals for the instruction of poor mothers in infant and family care [17]. Having faced tremendous barriers to her medical education, Dr. Elizabeth Blackwell wanted to open the door to the study of medicine for future women, who were still barred from admission by many medical institutions. Her dream materialized in 1868, when she and her sister, Dr. Emily Blackwell, founded one of the first four-year medical schools in the country - Women’s Medical College of the New York Infirmary. The college was adjacent to the infirmary such that students may attend lessons in medicine and then gain clinical training next door. Dr. Emily Blackwell served as the dean for many years, while Dr. Elizabeth Blackwell chose to teach as the professor of hygiene. The Women’s Medical College of the New York Infirmary became known for its excellence and rigorous curriculum. In the present day, the historic Women’s Medical College and New York Infirmary for Indigent Women and Children is still running as part of Weill Cornell Medicine and has been renamed the “New York Presbyterian Lower Manhattan Hospital” [5].

United Kingdom

**Figure 1:**
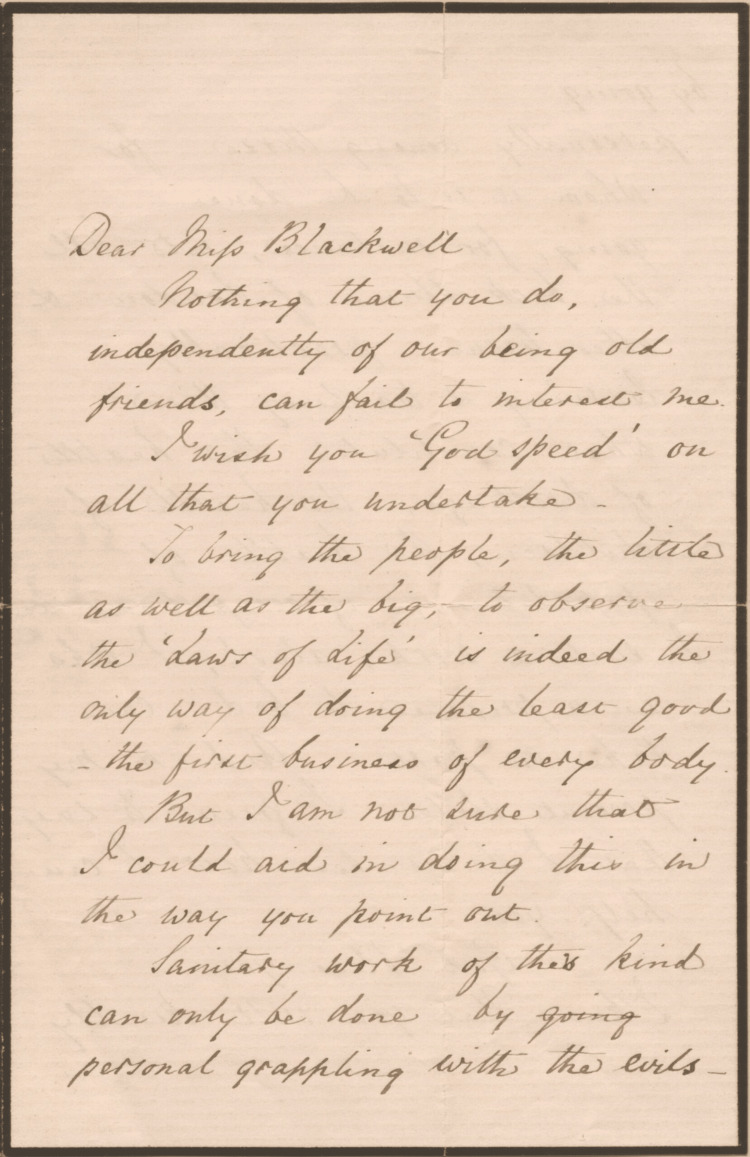
Letter from Florence Nightingale to Elizabeth Blackwell (August 2, 1871) "Nothing you do, independently of our being old friends, can fail to interest me." - Ms. Florence Nightingale to Dr. Elizabeth Blackwell The authors of this paper have obtained permission to reproduce this image from its original publishers. [18]

Never idle, Dr. Elizabeth Blackwell chose to leave the Medical School in the capable hands of her sister Dr. Emily Blackwell and moved back to London in 1858. In the UK, she hoped to collaborate with her old friend Ms. Florence Nightingale to start another women’s medical college. However, Ms. Florence Nightingale wished to start a nursing school with Dr. Elizabeth Blackwell as its superintendent. The two friends butted heads on their future endeavors despite their core purposes being one and the same - to help women advance in the field of medicine. However, Dr. Elizabeth Blackwell was adamant believing that women must endure equal requirements as men not to be re-relegated as the inferior sex once again, for which she made it her life's career to disprove. Ms. Florence Nightingale wrote to Dr. Elizabeth Blackwell, “You to educate a few highly cultivated [women] - I to diffuse as much knowledge as possible” (Figure 1).

Setting their differences aside, Ms. Florence Nightingale wrote a letter on Dr. Elizabeth Blackwell’s behalf to the President of the Medical Council, Sir Benjamin Brodie. Thanks to this connection, Dr. Elizabeth Blackwell became the first woman to be registered in the British Medical Registrar in 1859, a requirement to practice medicine there. Though both women held great respect and admiration for each other, the strong young friendship between Ms. Florence Nightingale and Dr. Elizabeth Blackwell was never the same. Dr. Elizabeth Garrett Anderson, who became England’s first female doctor was inspired by Dr. Elizabeth Blackwell [6].

**Figure 2:**
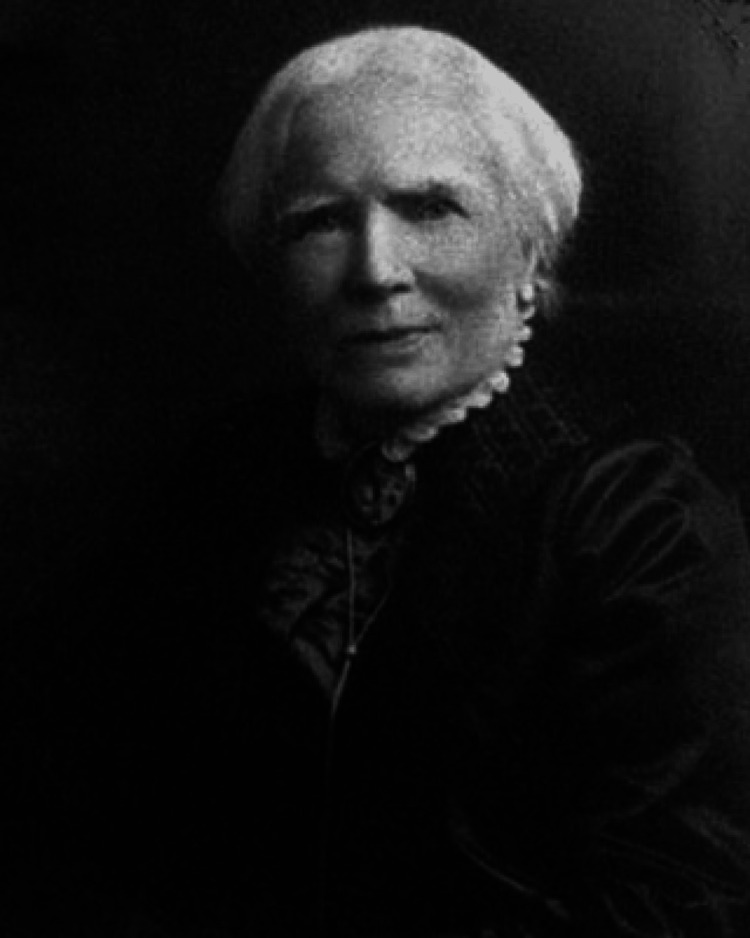
Dr. Elizabeth Blackwell The authors of this paper have obtained permission to reproduce this image from its original publishers. [25]

In the 1860s, Dr. Elizabeth Blackwell worked tirelessly to support the acceptance of women in medicine in the United Kingdom (UK). Not ready to give up clinical practice, Dr. Elizabeth Blackwell set up a private practice in London in the 1870s. She helped found the National Health Society (NHS) in 1871, to educate the public on hygiene and health, with its motto being "Prevention is better than Cure." She campaigned for the admission of women to medical universities in the UK, and in 1876 the legislation permitting women to earn their medical degree alongside men was passed. Doctors Elizabeth Blackwell, Sophia Jex-Blake, and Elizabeth Garrett Anderson began the London School of Medicine for Women in 1874 to prepare women for the Apothecaries Hall licensing examinations. There, she taught students as a professor of gynecology. Dr. Elizabeth Blackwell wrote and published countless books and writings on various medical topics throughout her life. Over the later forty years of her life, she was an active participant in many reform movements including moral reform, sexuality, hygiene, medical education, preventative medicine, women's suffrage, government morals, and the abolition of prostitution and slavery (Figure 2) [1, 19-24].

American Civil War, 1861: US Sanitary Commission

When the American Civil War broke out, Dr. Elizabeth Blackwell established a nurse training association to provide medical care and services on the battlefield. She was inspired by and learned from the efforts of her friend, Ms. Florence Nightingale, during the Crimean War. President Abraham Lincoln would eventually approve this association into becoming the United States Sanitary Commission. Dr. Elizabeth Blackwell was a guest of the White House, where she met President Lincoln.

Much like anyone meeting a celebrity figure, Dr. Elizabeth Blackwell wrote excitedly to her daughter, Kitty, about her meeting with the President, “A tall, ungainly loose-jointed man was standing in the middle of the room. He came forward with a pleasant smile and shook hands with us” [3].

## Conclusions

Dr. Elizabeth Blackwell never relented in her support and activism toward a better future for society. She protected and taught the poor about healthy lifestyle practices, prevented disease through her campaigns and efforts for community health, and trail-blazed the path for women to enter the medical profession. As America’s first female physician and the first woman to be registered to practice medicine in the United Kingdom, Dr. Elizabeth Blackwell's legacy and service to medicine is unparalleled.
